# Effects and mechanisms of resistance training on corticospinal adaptation

**DOI:** 10.3389/fphys.2025.1569639

**Published:** 2025-06-26

**Authors:** Chengjun Liang, Honglin Liu

**Affiliations:** School of Physical Education, Liaoning Normal University, Dalian, China

**Keywords:** resistance training, corticospinal adaptation, corticospinal excitability, cortical inhibition, effect, mechanism

## Abstract

Resistance training has a wide range of applications in sports, national fitness, and sports rehabilitation. In the early stages of resistance training, muscle volume did not show significant changes, but strength increased significantly, mainly due to adaptive changes in the human nervous system. This article focuses on exploring the effects and mechanisms of resistance training on the human corticospinal central nervous system, aiming to provide a theoretical reference for the scientific design of resistance training programs in various fields. Resistance training can enhance corticospinal excitability, lower the threshold for active movement, and increase the motor-evoked potential with increasing resistance training intensity. Resistance training significantly reduced short interval cortical inhibition and shortened the duration of cortical silence. After resistance training, the active muscle recruitment curve area significantly increased. Resistance training reduces the degree of coactivation between the agonist and antagonist muscles, reduces the cortical inhibitory effect of the active muscle, and increases the cortical promoting effect. The mechanisms of excitatory changes in the central nervous system during resistance training mainly include corticospinal adaptation, reticulospinal tract adaptation, and spinal cord adaptation. These mechanisms are mainly achieved through increased synaptic connectivity of cortical spinal motor neurons or increased excitability of motor neurons, or through increased synaptic efficacy of projections from the reticulospinal to the spinal cord. The mechanism of cortical inhibition reduction mainly be achieved by sensory feedback reducing the excitability of cortical inhibition circuits or accompanying activation of cortical facilitation networks.

## 1 Introduction

The central nervous system plays a crucial role in motor practice, from the generation of muscle strength to the formation of motor skills (Maeo et al., 2021). Resistance training can cause adaptive changes in the nervous system, including cortical and subcortical adaptation, which can increase the activation of motor neurons and may contribute to an increase in muscle strength associated with training (Siddique et al., 2020). Resistance training can cause plasticity changes in the central nervous system (Mason et al., 2017a). Adaptation to resistance training in the cortex and subcortical regions can increase the activation of motor neurons and enhance neural drive, which is a contributing mechanism to the increase in muscle strength associated with training. From this perspective, resistance training may be a useful intervention measure that can be used clinically to regulate cortical circuits (Mason et al., 2019a). Therefore, understanding the central nervous system response to resistance training is an important part of understanding the strength development characteristics of numerous clinical exercise rehabilitation and healthy populations. This study mainly reviews the literature reports on the adaptive changes and mechanisms of resistance training in the central nervous system in recent years, in order to clarify the impact and mechanisms of resistance training on the central nervous system and provide some useful theoretical reference for the design of resistance training programs in competitive sports, national fitness, and sports rehabilitation.

## 2 Research methods

This article searched literature databases such as Google Scholar, PubMed, Springer, Elsevier, etc., regarding the effects of resistance training on the central nervous system. The study combined “resistance training” and its synonym “strength training” with “neural adaptations,” “motor evolved potential,” “cortical excitation,” “subcortical inhibition,” and “cortical responses. Analyze the literature retrieved from the perspective of the effects and mechanisms of resistance training on the central nervous system.

## 3 The effects of resistance training on corticospinal adaptation

### 3.1 Adaptive changes in corticospinal excitability

Transcranial magnetic stimulation (TMS) of the motor cortex is a safe and non-invasive technique that can quantitatively evaluate human cortical and corticospinal activity. The magnitude of motor evoked potential (MEP) induced by single-pulse transcranial magnetic stimulation (TMS) is the most common indicator measured in resistance training research, typically used to infer changes in the excitability of the primary motor cortex (M1). After short-term resistance training (2 weeks), there was a significant increase in corticospinal excitability (CSE) (Mason et al., 2020; Wilson et al., 2023). After rhythmic resistance training using a metronome (Leung et al., 2017), CSE increased by 40%, indicating that resistance training can significantly improve corticospinal excitability. The long-term resistance training group has a lower active motor threshold (AMT) at low output target forces (15% and 25% MVC) compared to the non-resistance-trained group, which may be due to increased excitability in certain areas of the descending corticospinal pathway, thereby increasing cortical motor drive to the training muscles and reducing the activation threshold of the cortical facilitation circuit after prolonged resistance training (Lahouti et al., 2019). Long-term resistance training also showed a significant increase in spinal excitability of non-dominant biceps at high output (50% and 70% MVC) (Philpott et al., 2015), indicating that long-term resistance training can alter cortical spinal excitability. The impact of different training intensities on cortical spinal excitability varies (Alibazi et al., 2022), and one study (David et al., 2019) have shown that as the training load intensity increases, the motor-evoked potential significantly increases. After acute high-intensity isometric resistance training, the excitability of corticospinal axons was also promoted (Nuzzo et al., 2016).

However, a study shows that there is no correlation between increased muscle strength and changes in corticospinal excitability after long-term resistance training (Tallent et al., 2013). Some studies found that resistance training had no effect on enhancing the excitability of corticospinal axons. For example, after 4 weeks of high-intensity, isometric resistance training with increasing load, there was no change in cervicomedullary motor evoked potential (CMEP), indicating that corticospinal transmission and motor neuron excitability was not affected by training (Nuzzo et al., 2017). The lack of changes in cervicomedullary motor evoked potentials indicates that training neither affects corticospinal transmission nor motor neuron excitability. Therefore, the increase in muscle strength after resistance training is not due to an increase in the excitability of the corticospinal axons. On the contrary, it seems to reflect an adaptation that reduces corticospinal suppression and increases spinal nerve drive at the cortical level (Siddique et al., 2020).

These inconsistent views may be due to different types of resistance training (such as isometric and dynamic resistance training) and different muscle working conditions during training (such as near and far fixed muscle training) resulting in inconsistent outcomes. Inconsistent measurement methods are also one of the reasons for inconsistent results. For example, MEP data measurement may vary depending on the TMS intensity used in different studies, or may involve inconsistent muscle states during MEP measurements, such as neurophysiological measurements taken during rest or background muscle activity. Some studies have used different muscle contraction intensities during muscle activity measurements.

### 3.2 Adaptive changes in cortical inhibition

Short interval cortical inhibition (SICI) is typically measured using paired-pulse TMS and is commonly used to evaluate the inhibitory effect of the cerebral motor cortex and monitor changes in cortical motor excitability after intervention (Hunter et al., 2016). Long-term resistance training significantly reduced SICI, indicating the existence of adaptive processes that inhibit and promote network activation, which may reduce SICI and increase cortical motor drive to training muscles after prolonged resistance training (Lahouti et al., 2019). Both acute resistance training (Leung et al., 2015) and short-term resistance training (Kidgell et al., 2015) interventions can lead to a reduction in SICI. These results indicate that corticospinal plasticity occurs throughout the entire training period, and repeated stimulation of strength training is sufficient to cause long-term changes in muscle strength and cortical plasticity (Mason et al., 2020).After 2 weeks of resistance training for the elderly, cortical inhibition was also lower (Christie and Kamen, 2014), which is consistent with the training performance of young people. This indicates that repeated high-intensity voluntary muscle activation in the form of short-term high-load strength training can reduce SICI.

Single pulse and dual pulse TMS of the primary motor cortex (M1) can be used to evaluate the excitability of inhibitory motor networks (Yacyshyn et al., 2016). The reduction of cortical silent period (CSP) duration is an important neural adaptation for resistance training (Manca et al., 2018). Strength training reduces the duration of CSP and decreases SICI. Therefore, strength training reduces the synaptic efficacy of inhibitory networks in M1 and corticospinal pathways, indicating a new neural adaptation to strength training. Resistance strength training can reduce corticospinal inhibition, targeting specific inhibitory neurons within the cortex that collectively increase the neural drive of the spinal motor neuron pool, thereby mechanically increasing muscle strength (Kidgell et al., 2017). This indicates that reducing corticospinal inhibition is important for increasing muscle strength.

However, there are also some studies whose conclusions do not support the above research results. After short-term (4-week 80% 1RM squat) resistance training, there was no change in the response to central nervous system stimulation compared to the first training, indicating that changes in the corticospinal characteristics of the vastus lateralis may not contribute to the increase in strength (Ansdell et al., 2020). This is similar to the results of Latella et al. (2016) showing no changes in SICI or long interval cortical inhibition (LICI) after strength training of the elbow flexors. Coombs et al. (2016) study also suggests that there is no significant difference between the resistance training group and the non-training group in terms of MEP, silent period (SP), and SICI. After different forms of resistance training, SICI showed a decreasing trend, but the difference was not significant (Gómez-Feria et al., 2023).

The differences in the literature may be due to differences in experimental control factors, including differences in muscle position during exercise (such as upper and lower limbs), characteristics of resistance training programs (such as movement speed rhythm, movement load, repetition frequency and number of sets, etc.), and experimental subjects. Studies have shown that different loads and training volumes (Nicholson et al., 2014), different movement speeds (Del Vecchio et al., 2019), different intervals (Scudese et al., 2015), and different estimation methods for each effect value (Gómez-Feria et al., 2023) can all affect the evaluation of neuromuscular performance in resistance training. The differences in the cortical circuits and corticospinal projections of neuromuscular responses among the monitored subjects may also be one of the reasons for the inconsistent results in the literature.

### 3.3 Effect of resistance training on recruitment curve

When studying the effects of resistance training on corticospinal activity, constructing a recruitment curve and comparing the overall gain of Area under the recruitment curve (AURC) may be a good method (Carson et al., 2013). AURC measurement broadly represents the postsynaptic state of subpopulations of pyramidal tract neurons activated by TMS (Ruddy et al., 2016), and its increase represents an increase in motor neuron recruitment (Mason et al., 2017b).

After high-load (80% 1RM) strength training, the AURC of MEP in both agonist and antagonist muscles significantly increased (Mason et al., 2019b). Only performing strength training on one limb resulted in an increase in the AURC of the contralateral homologous muscle (Mason et al., 2017a), which may represent a general increase in the excitability of M1 and neurons in the motor neuron pool. Leung’s study (Leung et al., 2017) suggests that using a metronome for strength training for 4 weeks resulted in a 40% increase in AURC. The increase in AURC after rhythmic strength training may be due to a decrease in corticospinal inhibition and an increase in sustained excitation between intracortical circuits.

### 3.4 Changes in corticospinal regulation of agonist and antagonist muscle coordination

Long-term resistance training gradually reduces the degree of coactivation of antagonist muscle, indicating that among muscles coordination may be the main long-term neural adaptation for resistance training (Balshaw et al., 2018). The changes in antagonist muscle behavior and the agonist corticospinal response jointly promote an increase in strength (Mason et al., 2019c). After 3 weeks of 80%1RM strength training, the corticospinal excitability of the biceps and wrist flexors increased, the duration of the silent period decreased, and the changes in corticospinal function were not related to the increase in muscle strength (Mason et al., 2017b). This indicates that the corticospinal response of proximal upper limb muscle strength training is the cause of changes in the connections between agonist and cooperative muscles related to strength production.

## 4 Mechanisms of corticospinal adaptation induced by resistance training

There are currently many studies exploring the mechanisms of adaptive changes in the Corticospinal caused by resistance training. The existing research mainly explores the central nervous system adaptation mechanism from the perspectives of Corticospinal drive and inhibition.

### 4.1 Mechanisms of adaptive changes in corticospinal nerve drive

Changes in corticospinal output during and after strength training may promote strength development by influencing motor unit behavior. The degree of muscle activation, such as the number of activated motor units and the firing rate of motor neurons, both change after strength training (Farina and Holobar, 2016). According to existing research, the adaptive mechanisms of corticospinal nerve drive induced by resistance training mainly include corticospinal adaptation, reticulospinal tract Adaptation, and spinal cord adaptation.

#### 4.1.1 Corticospinal adaptation

Given that motor units are controlled by inputs from the corticospinal tract to the motor neuron pool, changes in motor unit behavior may involve adaptive changes in the corticospinal tract from M1 to the spinal motor neuron pool. Among these possible adaptation sites, adaptation at the level above the spinal cord is the main region (Siddique et al., 2020). Another piece of evidence suggests that strength training increases voluntary activation without increasing cervicomedullary excitability (Nuzzo et al., 2017), all of which suggests that the regulation of M1 levels may be the reason for changes in motor unit behavior. Individuals undergoing long-term resistance training showed no changes in CMEP (Pearcey et al., 2014), indicating that long-term resistance training resulted in neural adaptation of the corticospinal pathway upstream of spinal motor neurons. Strength training of the elbow flexor muscle increases the net output of motor neuron projections to the training muscle, possibly through increased efficacy of corticospinal motor neuron synapses or increased excitability of motor neurons (Nuzzo et al., 2016), or through central nervous system attempts to reduce or avoid muscle fatigue responses during training (Latella et al., 2017). In the current study, an increase in CSE and a decrease in inhibitory inputs from the motor neuron pool resulted in changes in the recruitment and firing rates of motor units throughout the entire strength training period, ultimately leading to an increase in the strength generated by muscle contraction. These corticospinal responses may reflect an increased ability of M1 to recruit and release motor units to the greatest extent possible, which may be achieved through an increase in input-output characteristics of corticospinal tracts after strength training (i.e., changes in CSE and silent period AURC), or through the release of SICI.

#### 4.1.2 Reticulospinal tract adaptation

The reticulospinal tract plays an important role in the growth of strength, and long-term strength training induces neural adaptation within the reticulospinal tract (Akalu et al., 2024). Animal experiments have shown that in primates, the majority of the descending drive force for motoneurons producing voluntary movement comes from the reticulospinal tract, not the corticospinal tract (Tapia et al., 2022). The research result of Glover and Baker (2020) on the neural adaptation mechanism of non-human primates after resistance training also indicates that the reticulospinal tract contributes greatly to neural adaptation after resistance training. Research on neuromuscular responses to acute and short-term (4-week) squat resistance training has shown that there are no changes in cortical, corticospinal, or spinal responses in specific and non-specific tasks (Ansdell et al., 2020). Climbers undergoing resistance training have a greater input to the reticulospinal tract of alpha motor neurons, which may be mediated by the adaptive effects of the reticulospinal tract triggered by long-term climbing and climbing-specific resistance exercise (Colomer‐Poveda et al., 2023). These researches suggest that the site of neural adaptation for resistance training may occur in the reticular formation (Atkinson et al., 2022). The above researches support the view that long-term resistance training can enhance the response of spinal motoneurons to input and generate corresponding output, thereby increasing strength. This is mainly achieved by increasing the synaptic function of the reticulospinal projection to the spinal cord. Therefore, the adaptability of spinal motoneurons within the reticulospinal pathway may contribute to the changes induced by long-term resistance training.

#### 4.1.3 Spinal cord adaptation

A study suggests that changes in corticospinal excitability after resistance training may be regulated downstream of the primary motor cortex (M1) (Mason et al., 2019a). In addition, an acute training study using transcranial electrical stimulation and cervicomedullary stimulation showed that strength training can alter the functional characteristics of spinal circuits and increase spinal excitability, but it does not affect the tissue of M1 (Nuzzo et al., 2016). Although the origin of changes in corticospinal excitability is currently unclear, the increase in spinal cord excitability after heavy load strength training indicates that the changes in corticospinal excitability are at least partially due to the increase in spinal cord excitability. After 4 weeks of resistance training, there was a significant increase in corticospinal excitability, but after continued training for 2 weeks (6 weeks later), there was no significant increase in corticospinal excitability,and throughout the entire training period, there was also no significant change in cortical inhibition (Wilson et al., 2023). The changes in Lumbar-evoked potential amplitude between young and elderly individuals may indicate that spinal cord adaptation is the main site of strength training in both young and elderly individuals (Gomez-Guerrero et al., 2024). These results suggest that the adaptation of the corticospinal tract caused by resistance training may not be due to changes in M1, but rather the result of adaptive changes in other parts of the corticospinal tract such as the spinal cord.

### 4.2 Mechanism of cortical inhibition reduction

There is a correlation between reducing cortical inhibition and muscle strength (Farina and Holobar, 2016), while the late stage of cortical silence seems to be more correlated with cortical inhibition (Yacyshyn et al., 2016). Therefore, the changes in the duration of the silence period may originate from the cortex or spinal cord, and eliminating this inhibition will help increase the downward driving force. Long-term resistance training can significantly reduce SICI (Lahouti et al., 2019). Resistance training reduces the excitability of the cortical inhibitory circuit, leading to a decrease in SICI. This may be due to the sensory feedback caused by muscle contraction, thereby reducing cortical inhibition (Vie et al., 2013). Resistance training with rhythm control can better enhance the adaptability of the corticospinal and reduce SICI (Gómez-Feria et al., 2023; Gordon et al., 2024), indicating that sensory feedback in resistance training can bring additional benefits to the adaptive changes of the central nervous system. After a single heavy load strength training, the SICI immediately decreased, but remained unchanged after the subsequent light load strength training. This indicates that strength training can be considered a form of motor learning at least under high loads, possibly due to the sensory feedback involved (Mason et al., 2019b). Although the exact mechanism by which SICI decreases with increasing muscle contraction intensity is not fully understood, one study (Brownstein et al., 2018) suggests that this may be due to the accompanying activation of the cortical facilitation network.

## 5 Conclusion

Although there is no unified view on the adaptation of the corticospinal in resistance training, the differences in training parameter characteristics (such as movement speed rhythm, movement load, number of repetitions, interval time, etc.) and experimental subjects in existing resistance training programs may be the main factors leading to differences in results. However the mainstream view is that resistance training can cause positive adaptive changes in the corticospinal of the central nervous system: resistance training enhances the excitability of the corticospinal, reduces the activation threshold, shortens the cortical quiet period, and reduces cortical inhibition. The mechanism of corticospinal adaptation in resistance training is mainly reflected in: 1) Resistance training causes adaptive changes in the cortex, thereby increasing the efficacy of corticospinal motor neuron synapses. 2) The reticular formation undergoes adaptive changes, which in turn increases the synaptic efficacy projected onto the spinal cord. 3) Resistance training causes adaptive changes in the spinal cord, leading to increased excitability or functional changes in spinal circuits. 4) The main mechanism of decreased corticospinal suppression is sensory feedback of muscle contraction or accompanying activation of cortical facilitation networks. At present, the understanding of the adaptation mechanism of the corticospinal is not complete, and the role of the cortical inhibitory circuit in mediating force generation has not been fully clarified. Current research often only considers a single control factor, and there is currently a lack of dose-response relationship studies on the effects of combinations of various training parameters on Corticospinal adaptation.
